# Case Report: Complete metabolic responses to trastuzumab–deruxtecan in HER2-altered solid tumors: two illustrative cases

**DOI:** 10.3389/fimmu.2026.1752694

**Published:** 2026-04-17

**Authors:** Coline Le Meur, Pierre-Yves Le Roux, Pierre Alemany, Frédéric Chauvelot, Olivier Pradier, Clémence Niel, Alex Bellange, Christos Chouaid, Christophe Massard, Karim Amrane

**Affiliations:** 1 Department of Radiotherapy, University Hospital of Brest, Brest, France; 2 Department of Nuclear Medicine, University Hospital of Brest, Brest, France; 3 UMR Inserm 1304 GETBO, IFR 148, Université de Bretagne Occidentale, Brest, France; 4 Department of Pathology, Ouest Pathologie Brest, Brest, France; 5 Department of Pharmacy, Regional Hospital of Morlaix, Morlaix, France; 6 Department of Oncology, Regional Hospital of Morlaix, Morlaix, France; 7 Department of Pulmonology, Centre Hospitalier Intercommunal (CHI) Créteil, Créteil, France; Institut Mondor de Recherche Biomédicale, U955 Inserm-Université Paris Est Créteil, Créteil, France; 8 Département d’Innovation Thérapeutique et des Essais Précoces (DITEP), Gustave Roussy, Université Paris Saclay, Villejuif, France

**Keywords:** FDG-PET/CT, HER2 exon 20 mutation, HER2 expression, NSCLC, trastuzumab-deruxtecan, urothelial carcinoma

## Abstract

**Background:**

Human epidermal growth factor receptor-2 (HER2) alterations, including activating mutations and gene amplification, are emerging therapeutic targets in several solid tumors. Trastuzumab–deruxtecan (T-DXd), a HER2-directed antibody-drug conjugate (ADC), has demonstrated antitumor activity across multiple HER2-altered cancers. However, complete metabolic responses (CMRs) remain uncommon, particularly when assessed by 2-deoxy-2-[18F] fluoro-D-glucose positron emission tomography-computed tomography (FDG-PET/CT).

**Presentation of cases:**

We report two patients with metastatic HER2-driven malignancies who achieved a CMR under T-DXd. The first case concerns a patient with metastatic non-small cell lung cancer harboring a HER2 exon 20 insertion who progressed on chemoimmunotherapy and HER2-directed antibodies. Third-line T-DXd led to rapid metabolic improvement and complete resolution of all lesions, including a brain metastasis. The second case involves a patient with metastatic micropapillary urothelial carcinoma with HER2 expression, refractory to platinum chemotherapy, avelumab maintenance, and enfortumab vedotin. T-DXd induced an early partial metabolic response (PMR), followed by a confirmed CMR after four cycles.

**Conclusion:**

These two observations illustrate the capacity of T-DXd to induce deep and complete metabolic remissions in distinct HER2-altered solid tumors. They support further development of HER2-targeted ADCs beyond traditional indications and highlight the value of FDG-PET/CT for assessing the depth of response to these agents.

## Introduction

Human epidermal growth factor receptor-2 (HER2) is a member of the epidermal growth factor receptor (EGFR) family and activates key oncogenic pathways including MEK–ERK and PI3K–AKT. Activating mutations, amplifications, or overexpression promote constitutive dimerization and uncontrolled tumor proliferation (1). HER2 alterations represent important oncogenic drivers across multiple solid tumors. Beyond breast and gastric cancers, HER2 alterations have been identified in non–small cell lung cancer (NSCLC), urothelial carcinoma (UC), and several other malignancies, supporting the development of HER2-targeted therapies in a tumor-agnostic manner (1, 8).

The discovery of actionable molecular alterations in solid tumors has led to major therapeutic advances, particularly with tyrosine kinase inhibitors (TKIs) (2). Among these oncogenic drivers, aberrations of HER2 represent a therapeutic challenge. Although HER2 mutations occur in 2%–4% of NSCLC (3), most frequently presenting as exon 20 in-frame insertions that are mutually exclusive of other oncogenic events (4, 5), no HER2-targeted therapy has been formally approved in this setting. Similarly, HER2 expression in UC ranges widely from 6% to 80% (6) and is associated with aggressive histology, higher metastatic potential, and poorer prognosis (7, 8).

Trastuzumab–deruxtecan (T-DXd), an antibody-drug conjugate (ADC), demonstrated durable antitumor activity in HER2-mutant NSCLC in the DESTINY-Lung01 trial, although complete responses (CRs) remained exceptional (1/91 patients) (9). Similarly, the DESTINY-PanTumor02 trial showed promising activity in HER2-expressing UC, but CRs were rare (1/41 patients) (10), and none have been reported on FDG-PET/CT evaluation. To date, only one FDG-PET/CT-assessed complete metabolic response (CMR) has been published in HER2-mutant NSCLC (11). Consequently, real-world observations of deep responses may provide valuable insights into the therapeutic potential of T-DXd in HER2-driven malignancies.

2-deoxy-2-[18F] fluoro-D-glucose positron emission tomography-computed tomography (FDG-PET/CT) is a key imaging modality for the staging and therapeutic monitoring of solid tumors. It allows detection of metastatic spread with high sensitivity and specificity in non-small cell lung cancer (NSCLC) (12–14) and is increasingly used to evaluate treatment response in tumors harboring oncogenic drivers, including urothelial carcinoma (UC) (15). Beyond conventional staging, FDG-PET/CT has been progressively integrated into clinical trials and therapeutic evaluation strategies, particularly in the context of targeted therapies (16). Metabolic response assessment using PET-based criteria, such as the PET Response Criteria in Solid Tumors (PERCIST), may provide complementary information to conventional anatomical criteria like RECIST, allowing earlier and more sensitive evaluation of treatment efficacy (17, 18). Recent studies further support the growing role of FDG-PET/CT in patient selection and response monitoring within precision oncology trials (19).

Here, we report two distinct cases, one of metastatic NSCLC with a HER2 exon 20 mutation, and one of metastatic HER2-expressing UC, both achieving a complete metabolic response (CMR) under T-DXd, assessed by FDG-PET/CT according to PERCIST.

## Description of cases

**Figure 1:**
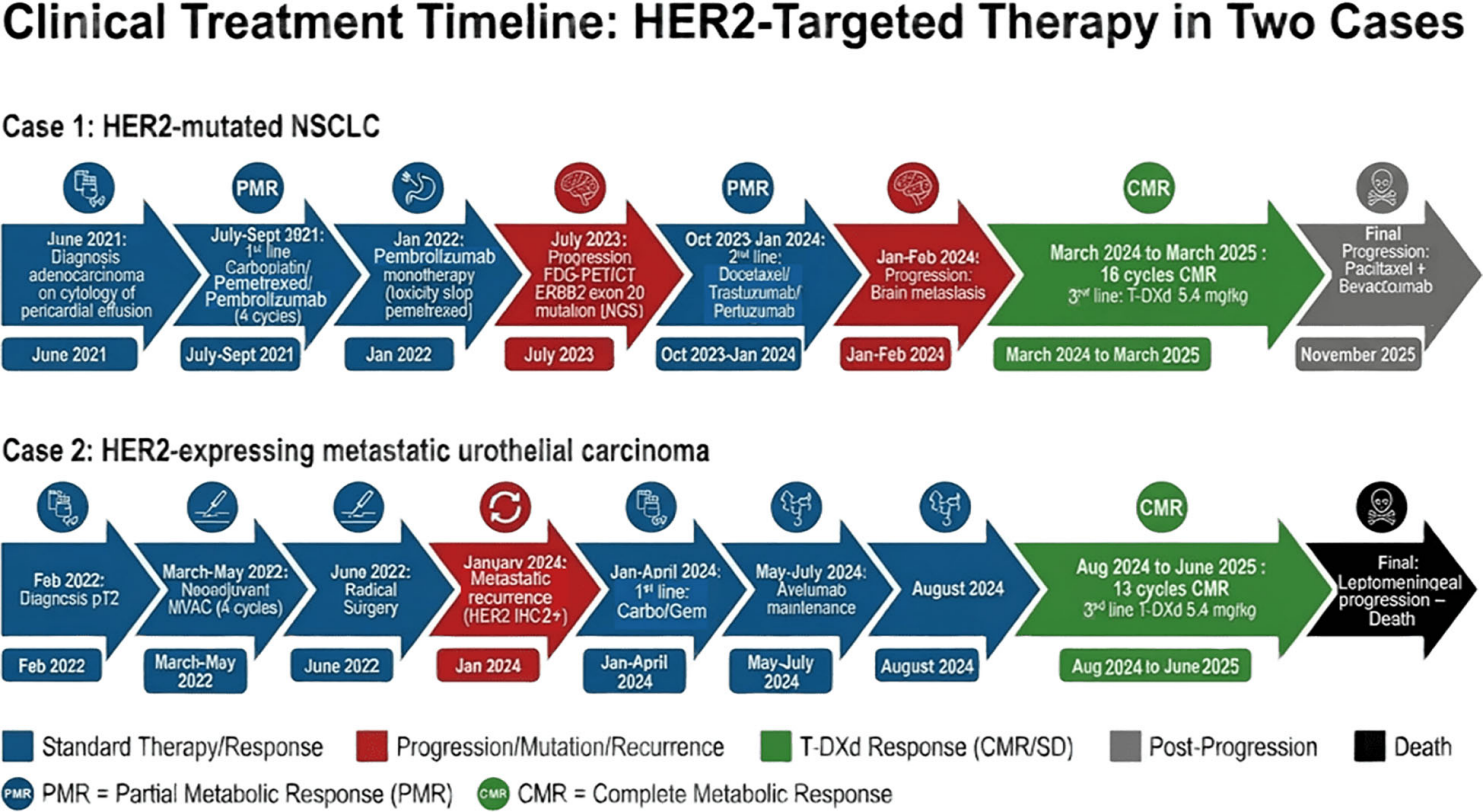
Clinical timelines of the two reported cases. **(A)** Case 1: HER2-mutated metastatic non-small cell lung cancer treated with chemoimmunotherapy, HER2-directed antibodies, and trastuzumab–deruxtecan (T-DXd), leading to a complete metabolic response. **(B)** Case 2: HER2-expressing metastatic urothelial carcinoma treated with platinum-based chemotherapy, avelumab maintenance, enfortumab vedotin, and trastuzumab–deruxtecan, resulting in a complete metabolic response.

The clinical course and treatment timelines of both patients are summarized in **Figure 1**.

### Case 1: HER2-mutated metastatic NSCLC

A 76-year-old non-smoker man with a history of arterial hypertension and atrial flutter was diagnosed with PD-L1 < 1% lung adenocarcinoma on cytology of pericardial effusion (**Figure 1A**). Initial CT imaging showed a right upper lobe primary tumor with multiple pulmonary metastases. Brain imaging revealed two osteolytic lesions of the right and left paramedian occipital bone without cortical destruction and considered non-specific.

First-line treatment consisted of carboplatin (AUC 5), pemetrexed (500 mg/m²), and pembrolizumab (200 mg) administered every 3 weeks from July to September 2021 (four cycles). Maintenance therapy with pemetrexed and pembrolizumab was initiated in October 2021. Pemetrexed was discontinued in January 2022 due to treatment-related toxicity including nausea, lower limb edema, and asthenia, while pembrolizumab was continued as monotherapy, resulting in a partial metabolic response (PMR).

**Figure 2:**
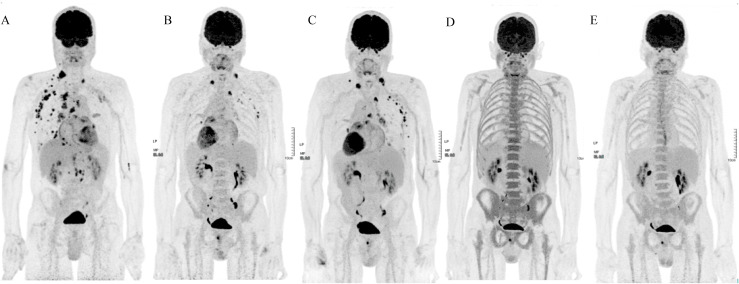
FDG-PET/CT findings in Case 1 (HER2-mutated metastatic non-small lung cancer) during the course of treatment. **(A)** Baseline FDG-PET/CT before second-line therapy showing hypermetabolic involvement of supradiaphragmatic and infradiaphragmatic lymph nodes, pleural lesions, and right lung parenchymal progression (MIP). **(B)** Partial metabolic response (PMR) under docetaxel–trastuzumab–pertuzumab, with decreased uptake in thoracic and abdominal disease sites (MIP). **(C)** Metabolic progression under trastuzumab–pertuzumab maintenance, with increased activity in thoracic lesions and new distant metastases (MIP). **(D)** Early PMR after initiation of trastuzumab-deruxtecan (T-DXd), with marked reduction of FDG uptake and disappearance of previously identified intracranial disease (MIP). **(E)** Complete metabolic response (CMR) on FDG-PET/CT, with full resolution of all previously hypermetabolic lesions (MIP).

**Figure 3:**
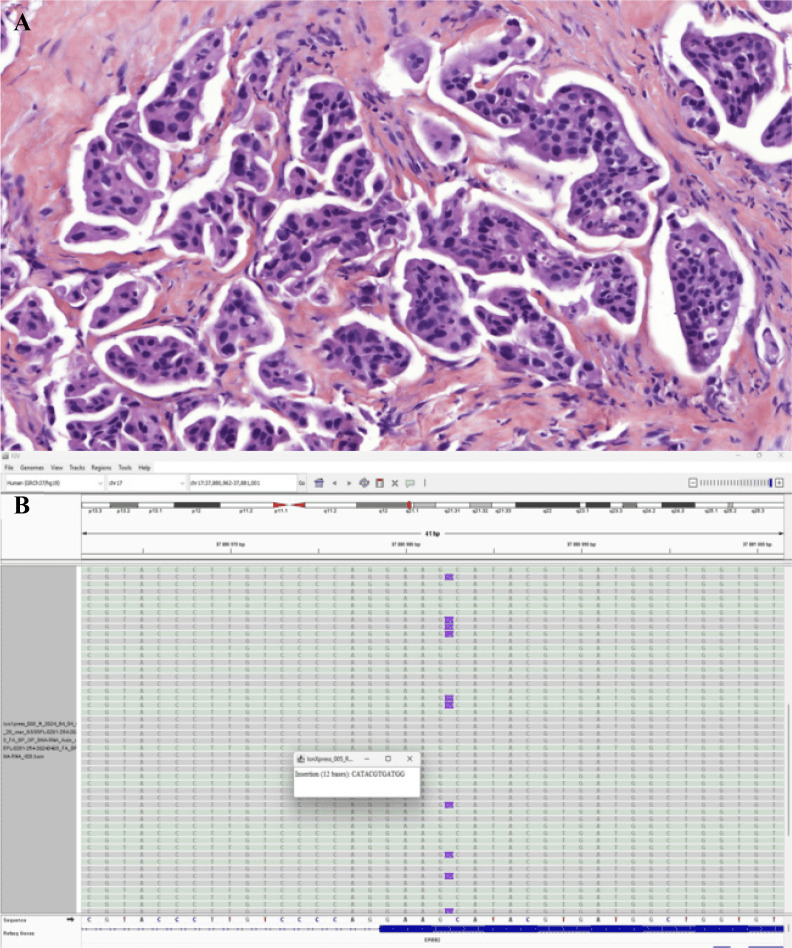
Histopathological and molecular characterization of Case 1 (HER2-mutated NSCLC). **(A)** Hematoxylin–eosin (H&E) staining confirming lung adenocarcinoma morphology on repeat biopsy. **(B)** Molecular detection of an ERBB2 exon 20 insertion mutation (p.Tyr772_Ala775dup) identified by targeted DNA-based next-generation sequencing (Oncomine™ Solid Tumor DNA panel and Oncomine™ Solid Tumor PLUS v2; Thermo Fisher Scientific) performed on the Ion GeneStudio™ S5 Prime System. Bioinformatic analysis was conducted using Ion Reporter™ software with the AmpliSeq Colon and Lung Cancer v2 pipeline.

In July 2023, FDG-PET/CT performed for worsening asthenia demonstrated metabolic progression with increased uptake in multiple supra- and subdiaphragmatic lymph nodes, right pleuro-pulmonary lesions, and a left lower lobe pulmonary focus (**Figure 2A**). A cervical lymph node biopsy performed on July 25, 2023 confirmed metastatic lung adenocarcinoma (**Figure 3A**). Molecular analysis was performed using targeted DNA-based next-generation sequencing (NGS) with the Oncomine™ Solid Tumor DNA panel and the Oncomine™ Solid Tumor PLUS v2 panel (Thermo Fisher Scientific, Waltham, Massachusetts, USA). Libraries were sequenced on the Ion GeneStudio™ S5 Prime System (Thermo Fisher Scientific), and bioinformatic analysis was performed using Ion Reporter™ software with the AmpliSeq Colon and Lung Cancer v2 pipeline. This analysis identified an ERBB2 exon 20 insertion mutation (p.Tyr772_Ala775dup) (**Figure 3B**). This mutation is a known activating HER2 exon 20 insertion recurrently reported in NSCLC and breast cancers (20, 21). Enrollment in a clinical trial was not possible due to the patient’s atrial flutter, which was an exclusion criterion.

**Figure 4:**
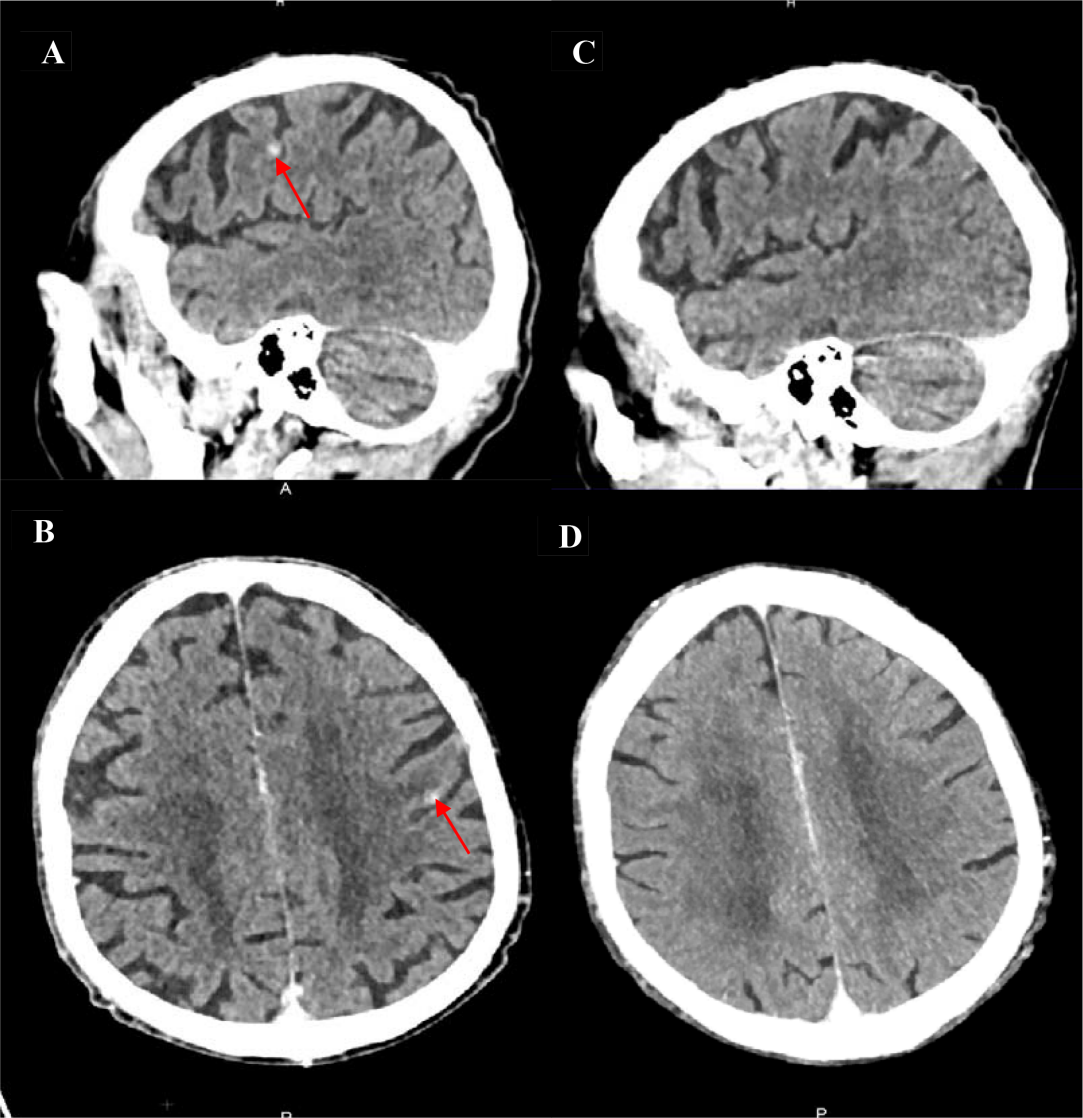
Brain involvement and response to trastuzumab–deruxtecan in Case 1. **(A, B)** Baseline brain CT showing a left parietal metastasis with contrast enhancement and perilesional edema; the red arrows highlight the metastatic lesion on both images (A Sagittal; B Axial). **(C, D)** Follow-up CT after the initiation of trastuzumab–deruxtecan demonstrating complete disappearance of the lesion previously indicated by red arrows (C Sagittal; D Axial).

Second-line therapy with docetaxel (75 mg/m²), trastuzumab (6 mg/kg), and pertuzumab (420 mg) was initiated in October 2023 following a loading dose (5, 22). Five cycles were administered until January 2024, leading to a PMR after cycles three and five (**Figure 2B**). The sixth cycle was not administered due to poor tolerance of docetaxel. Maintenance therapy with trastuzumab and pertuzumab was continued from January to February 2024 but was discontinued due to clinical deterioration marked by asthenia. FDG-PET/CT confirmed disease progression associated with the appearance of a new left parietal brain metastasis (**Figures 2C**, **4A, B**).

Third-line treatment with trastuzumab deruxtecan (T-DXd) was initiated in March 2024 at a dose of 5.4 mg/kg (345 mg) every 3 weeks. FDG-PET/CT showed an early PMR after three cycles with the disappearance of the brain metastasis (**Figures 2D**, **4C, D**). Treatment was continued for approximately 1 year (16 cycles), with serial FDG-PET/CT and brain imaging performed every 3 months. Thoracic CT scans were performed regularly (every 6 weeks) to monitor potential interstitial lung disease, and cardiac monitoring, including assessment of left ventricular ejection fraction (LVEF), conducted every 3 months.

A CMR was achieved and maintained for approximately 12 months (**Figure 2E**). Disease progression eventually occurred, and the patient subsequently received paclitaxel (at a dose of 90 mg/m² on days 1, 8, and 15 every 28 days) combined with bevacizumab (at a dose of 15 mg/kg on days 1 and 15 every 28 days).

### Case 2: HER2-expressing metastatic urothelial carcinoma

A 71-year-old man, a former smoker, was diagnosed in February 2022 with muscle-invasive urothelial carcinoma of the bladder (pT2) (**Figure 1B**). The patient received neoadjuvant dose-dense MVAC chemotherapy (methotrexate, vinblastine, doxorubicin, and cisplatin) between March and May 2022 (four cycles), followed by radical surgery in June 2022. Pathological examination confirmed urothelial carcinoma staged pT2N3M0.

After 18 months of surveillance, metastatic recurrence was diagnosed in January 2024 with bone and lymph node involvement confirmed histologically. Molecular analysis using a targeted RNA-based NGS panel revealed no actionable alterations. Immunohistochemistry demonstrated HER2 expression with moderate membranous staining in ≥10% of tumor cells [HER2 IHC 2+ according to ASCO/CAP 2018 guidelines (23)].

First-line systemic therapy for metastatic disease consisted of carboplatin (AUC 5 on day 1) and gemcitabine (1,000 mg/m² on days 1 and 8, every 21 days) administered between January and April 2024 (four cycles). Maintenance therapy with avelumab (10 mg/kg, 700 mg) was subsequently initiated between May 2024 and July 2024 but was discontinued after six infusions due to disease progression with increased lymph node and bone metastases.

**Figure 5:**
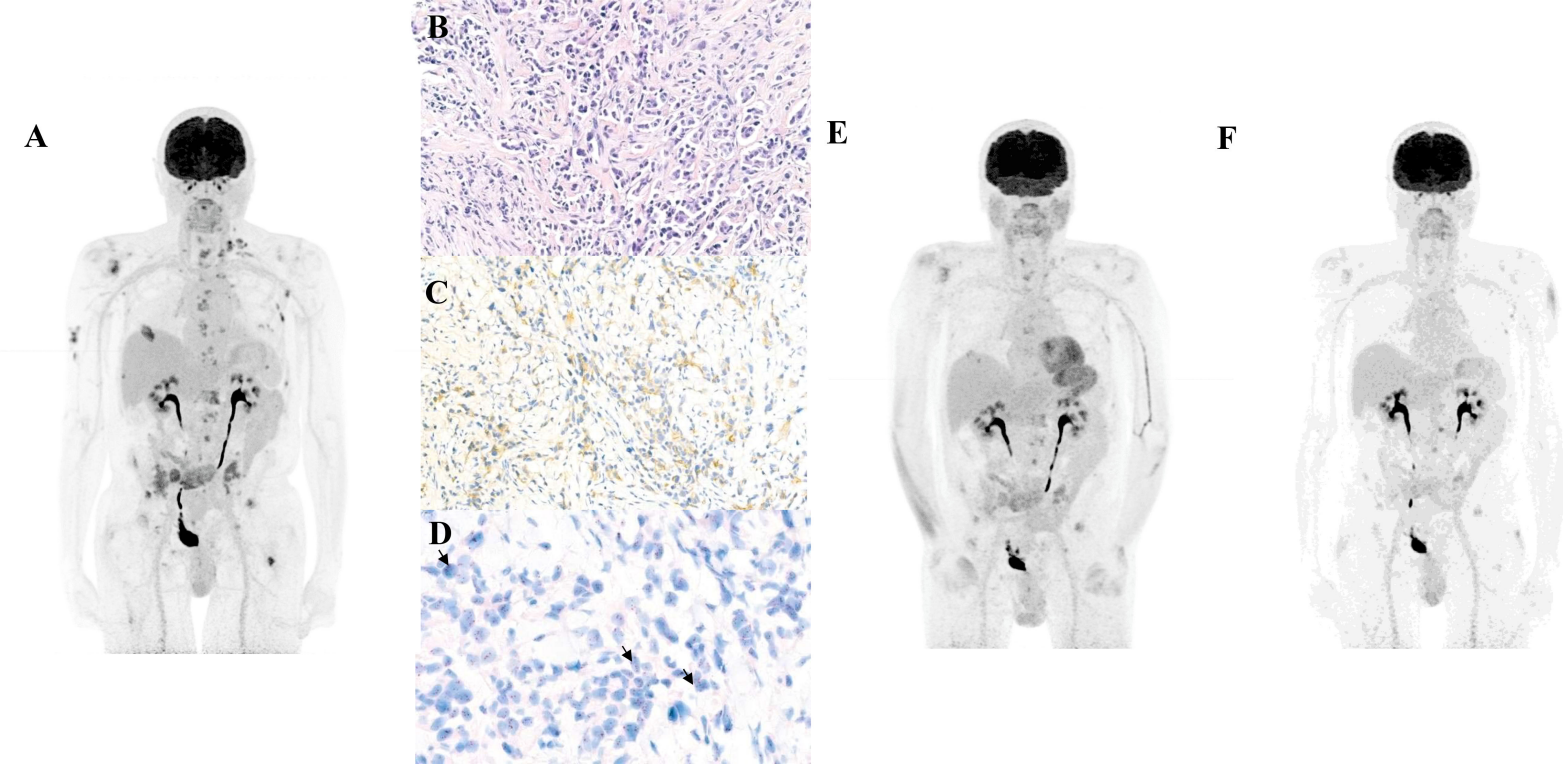
Imaging and molecular features of Case 2 (HER2-expressing urothelial carcinoma). **(A)** FDG-PET/CT showing metabolically active supradiaphragmatic lymph nodes and bone metastases consistent with progression (MIP). **(B)** HER2 immunohistochemistry showing membranous staining in ≥10% of tumor cells, corresponding to HER2 IHC 2+ according to ASCO/CAP 2018 guidelines. **(C)** Higher-magnification HER2 membranous staining by immunohistochemistry (IHC) confirming moderate circumferential staining (HER2 IHC 2+). **(D)** Dual *in situ* hybridization (DISH) demonstrating chromosome 17 triploidy with ERBB2 amplification; the arrows indicate amplified ERBB2 signals. The HER2/CEP17 ratio was 0.82 (54 HER2 signals/66 chromosome 17 signals counted in 20 tumor cells). **(E)** Partial metabolic response (PMR) after two infusions of trastuzumab–deruxtecan (MIP). **(F)** Complete metabolic response after four infusions (MIP). .

Second-line therapy with enfortumab vedotin was administered from May to July 2024 at a dose of 1.25 mg/kg on days 1, 8, and 15 every 28 days. After three cycles, FDG-PET/CT showed further disease progression with increased metabolic activity in supradiaphragmatic lymph nodes and bone lesions (**Figure 5A**).

In the absence of an available clinical trial, third-line treatment with trastuzumab–deruxtecan (T-DXd) was initiated in August 2024 at a dose of 5.4 mg/kg (345 mg) every 3 weeks. FDG-PET/CT demonstrated a rapid PMR after two cycles (**Figure 4E**), followed by a complete metabolic response after four cycles (**Figure 5F**). Treatment was continued for approximately 10 months (13 cycles) with regular FDG-PET/CT and brain imaging every 3 months. Thoracic CT scans were performed every 6 weeks to monitor for interstitial lung disease, and cardiac evaluation including assessment of left ventricular ejection fraction (LVEF) was conducted every three months.

After approximately 10 months of disease control, the patient developed meningeal progression presenting with oculomotor paralysis. Initial brain MRI was negative, but cerebrospinal fluid analysis confirmed leptomeningeal involvement. Molecular testing did not identify fibroblast growth factor receptor (FGFR) alterations. The patient’s clinical condition rapidly deteriorated, ultimately leading to death.

### Safety

For both patients, treatment with trastuzumab–deruxtecan was generally well tolerated. No grade ≥3 treatment-related adverse events according to Common Terminology Criteria for Adverse Events (CTCAE) version 5.0 were observed. Importantly, no interstitial lung disease or cardiotoxicity occurred during T-DXd treatment. The main adverse events were mild nausea and vomiting, which were effectively managed with supportive care.

## Discussion

T-DXd is a next-generation HER2-directed ADC whose unique structural and functional properties explain its remarkable activity in HER2-altered malignancies. The two cases we report, a HER2 exon 20-mutant NSCLC and a HER2-expressing metastatic UC, both achieving CMR on FDG-PET/CT, highlight the potential of T-DXd to induce deep and durable tumor eradication across distinct oncological contexts. Such CMRs remain exceptionally rare in the literature for both diseases.

T-DXd combines a humanized anti-HER2 IgG1, a cleavable tetrapeptide linker, and a highly potent membrane-permeable topoisomerase I inhibitor payload (DXd), delivered at a high drug-to-antibody ratio (DAR ≈ 8:1) (24). This configuration enables efficient HER2-dependent internalization, lysosomal cleavage of the linker, and intracellular liberation of DXd, which subsequently diffuses into the nucleus to induce topoisomerase-I–mediated DNA damage (25, 26). The membrane permeability of DXd allows diffusion beyond the initially targeted cell, driving bystander killing in tumors with heterogeneous HER2 expression (27, 28), a hallmark of both HER2-mutant NSCLC and HER2-positive UC.

Preclinical studies have demonstrated that HER2-directed ADC efficacy depends not only on receptor abundance but also on HER2 ubiquitination and internalization, which promote lysosomal trafficking and efficient payload release (29). This may be particularly relevant in HER2 exon 20-mutant NSCLC, where activating mutations favor accelerated receptor internalization compared with mere HER2 overexpression. ADC switching strategies, notably from T-DM1 to T-DXd, have been shown to overcome resistance through optimized linker cleavage and a more potent cytotoxic payload (26). These mechanisms align closely with our first case, where trastuzumab–pertuzumab double blockade failed, yet T-DXd induced rapid disease control and ultimately CMR.

In UC, HER2 overexpression and gene amplification are associated with aggressive behavior and poor prognosis, but actionable therapeutic options remain limited (30, 31). Despite a lack of large dedicated clinical studies, our second case illustrates that HER2-expressing UC may exhibit profound susceptibility to T-DXd when HER2 expression is sufficient to mediate effective ADC internalization. This observation is consistent with early pan-tumor data (9) and supports further evaluation of HER2-targeted ADCs in UC. Recent therapeutic developments further highlight the expanding role of HER2-targeted ADCs in UC. Notably, the combination of the HER2-directed ADC disitamab vedotin with the PD-1 inhibitor toripalimab has demonstrated significant clinical benefit in patients with HER2-expressing advanced urothelial cancer. In a recent randomized phase 3 trial, this combination significantly improved progression-free survival (13.1 vs. 6.5 months) and overall survival (31.5 vs. 16.9 months) compared with platinum-based chemotherapy, with an objective response rate exceeding 70% (32). These findings underscore the rapid evolution of HER2-targeted strategies in UC and reinforce the clinical relevance of targeting HER2-altered tumors with next-generation ADCs.

Importantly, T-DXd may also exert immune-stimulatory effects, including the release of damage-associated molecular patterns (DAMPs), activation of innate immune pathways (TLR4/STING), Fcγ-receptor engagement, and enhanced antigen presentation, potentially amplifying antitumor immunity (33, 34) in a manner particularly relevant for immunologically active tumors such as UC or NSCLC. This could partially contribute to the depth of response observed in both patients.

Complete responses with T-DXd remain uncommon in clinical trials. In the DESTINY-Lung02 study, complete responses were observed in a small minority of patients with HER2-mutant NSCLC. Similarly, CR rates were low in the DESTINY-PanTumor02 trial that evaluated multiple HER2-expressing solid tumors. Therefore, the complete metabolic responses observed in our two heavily pretreated patients highlight the potential depth of response achievable with T-DXd in selected HER2-driven malignancies.

Sequential use of ADCs is generally associated with limited efficacy due to potential cross-resistance mechanisms. However, in our second case, a complete metabolic response was achieved with T-DXd despite prior treatment with enfortumab vedotin. This observation is particularly noteworthy given the rapidly evolving therapeutic landscape in urothelial carcinoma, where both Nectin-4–targeted and HER2-targeted ADCs are under investigation. It also raises the clinically relevant question of optimal sequencing between these agents in HER2-amplified bladder cancer.

Finally, the remarkable CMRs observed in our two cases likely reflect efficient HER2-mediated internalization of the ADC, enabling optimal intracellular delivery of the DXd payload. Conversely, HER2 blockade alone is frequently insufficient due to the frequent co-activation of MAPK and PI3K/AKT pathways (≈44%) (35), which maintain downstream proliferation signals despite upstream receptor inhibition. Thus, ADCs, by bypassing simple receptor blockade and delivering cytotoxic payloads intracellularly, represent a rational and mechanistically superior strategy in HER2-driven tumors.

This report has inherent limitations related to its observational nature and the small number of patients. Nevertheless, the depth and duration of metabolic responses observed highlight the potential activity of T-DXd in HER2-driven tumors and warrant further investigation in prospective studies. FDG-PET/CT may be particularly valuable for evaluating early metabolic response to ADCs, which may precede anatomical tumor shrinkage.

These observations collectively underscore the relevance of T-DXd beyond traditional HER2-positive breast cancer, demonstrating compelling activity in HER2-mutant NSCLC and HER2-expressing UC. They support broader investigation of T-DXd in HER2-altered solid tumors and highlight the utility of FDG-PET/CT for monitoring deep responses in precision oncology, including complete metabolic remissions.

## Data Availability

The original contributions presented in the study are included in the article/supplementary material. Further inquiries can be directed to the corresponding author.
